# A comparison of five sets of overlapping and non-overlapping sliding windows for semen production traits in the Thai multibreed dairy population

**DOI:** 10.5713/ab.23.0230

**Published:** 2023-11-01

**Authors:** Mattaneeya Sarakul, Mauricio A. Elzo, Skorn Koonawootrittriron, Thanathip Suwanasopee, Danai Jattawa, Thawee Laodim

**Affiliations:** 1 Department of Animal Science, Nakhon Phanom University, Nakhon Phanom, 48000, Thailand; 2 Department of Animal Sciences, University of Florida, Gainesville, FL 32611-0910, USA; 3 Department of Animal Science, Kasetsart University, Bangkok 10900, Thailand; 4 Department of Animal Science, Kasetsart University, Kamphaeng Saen Campus, Nakhon Pathom 73140, Thailand

**Keywords:** Additive Genetic Variance, Biological Pathways, Dairy Cattle, Multibreed Population, Semen Production, Single Nucleotide Polymorphism Windows

## Abstract

**Objective:**

This study compared five distinct sets of biological pathways and associated genes related to semen volume (VOL), number of sperm (NS), and sperm motility (MOT) in the Thai multibreed dairy population.

**Methods:**

The phenotypic data included 13,533 VOL records, 12,773 NS records, and 12,660 MOT records from 131 bulls. The genotypic data consisted of 76,519 imputed and actual single nucleotide polymorphisms (SNPs) from 72 animals. The SNP additive genetic variances for VOL, NS, and MOT were estimated for SNP windows of one SNP (SW1), ten SNP (SW10), 30 SNP (SW30), 50 SNP (SW50), and 100 SNP (SW100) using a single-step genomic best linear unbiased prediction approach. The fixed effects in the model were contemporary group, ejaculate order, bull age, ambient temperature, and heterosis. The random effects accounted for animal additive genetic effects, permanent environment effects, and residual. The SNPs explaining at least 0.001% of the additive genetic variance in SW1, 0.01% in SW10, 0.03% in SW30, 0.05% in SW50, and 0.1% in SW100 were selected for gene identification through the NCBI database. The pathway analysis utilized genes associated with the identified SNP windows.

**Results:**

Comparison of overlapping and non-overlapping SNP windows revealed notable differences among the identified pathways and genes associated with the studied traits. Overlapping windows consistently yielded a larger number of shared biological pathways and genes than non-overlapping windows. In particular, overlapping SW30 and SW50 identified the largest number of shared pathways and genes in the Thai multibreed dairy population.

**Conclusion:**

This study yielded valuable insights into the genetic architecture of VOL, NS, and MOT. It also highlighted the importance of assessing overlapping and non-overlapping SNP windows of various sizes for their effectiveness to identify shared pathways and genes influencing multiple traits.

## INTRODUCTION

The Thai multibreed dairy population consists primarily of Holstein crossbred animals, along with small numbers of animals with various proportions of Brahman, Brown Swiss, Jersey, Red Dane, Red Sindhi, Sahiwal, and Thai Native [1]. A recent genome-wide association study (GWAS) on semen traits in this population identified specific single nucleotide polymorphisms (SNPs) associated with semen volume (VOL), number of sperm (NS), and sperm motility (MOT) across all 29 autosomes and the X chromosome [2]. This study revealed that these traits are influenced by genes involved in focal adhesion, actin cytoskeleton regulation, oxytocin signaling, axon guidance, B cell receptor signaling, rap1 signaling, and sphingolipid signaling pathways, which are closely linked to sperm morphology and physiology during spermatogenesis in Thai dairy bulls [2]. Similar research conducted on Holstein cattle in the United States identified gene sets associated with conception rate, involving small GTPases mediated signal transduction, neurogenesis, calcium ion binding, cytoskeleton, PI3K signaling in B lymphocytes, axonal guidance signaling, and role of macrophages pathways [3,4].

However, the utilization of individual SNPs in these studies may not represent the most efficient approach, because they offer limited genomic information specific to certain genomic regions [5,6]. Research in cattle by authors [7–10] reported using of haplotype for GWAS adds information more than obtained only by single SNPs. Therefore, an alternative strategy involves conducting haplotype association analyses based on overlapping windows, where contiguous neighboring SNPs are combined within a window for GWAS data analysis [11].

Previous studies employed sliding-window haplotypes of various sizes (3, 5, 7, 9, and 11 SNPs) in Nelore cattle [5] and multiple moving window sizes (3, 5, 7, and 9) in Han Chinese population [11]. The selection of an appropriate window size is critical because a larger window may encompass non-informative markers, whereas a smaller window may overlook informative markers [12]. However, the optimal window size and a standardized criterion for defining the optimal SNPs within each window size remain uncertain. Moreover, no studies have specifically investigated the optimal window size for identifying sets of SNPs associated with genes in biological pathways that influence semen traits in the Thai multibreed dairy population, nor have they explored a suitable strategy for selecting these window sizes. Therefore, the objective of this research was to compare five different sets of biological pathways and genes influencing VOL, NS, and MOT across overlapping and non-overlapping windows of various sizes (1, 10, 30, 50, and 100 SNP) in terms of their quantity, percentage, nomenclature, and functions in the Thai multibreed dairy population.

## MATERIALS AND METHODS

### Ethics approval

The dataset utilized in this study was obtained from cattle raised in commercial dairy farms that strictly adhere to the Good Agricultural Practices outlined by the National Bureau of Agricultural Commodity and Food Standards, as well as the Good Farming Management Practices mandated by the Department of Livestock Development, Ministry of Agriculture and Cooperatives, Thailand. Ethical clearance for conducting the study was granted by the Institutional Animal Care and Use Committee of Kasetsart University, with approval number ACKU60-AGR-009. The study was conducted in accordance with the ethical guidelines and regulations, ensuring the welfare and ethical treatment of the animals involved.

### Animals, management, and feeding

The dataset comprised 131 bulls with phenotypic records for VOL (n = 13,533), NS (n = 12,773), and MOT (n = 12,660) obtained from the Semen Production and Dairy Genetic Evaluation Center of the Dairy Farming Promotion Organization of Thailand (DPO). These bulls were the offspring of 62 sires and 112 dams. The sires of the bulls were associated with the Semen Production and Dairy Genetic Evaluation Center of the DPO, while the dams were from 87 dairy farms situated across the Central, Northeastern, Northern, and Southern regions of Thailand. The bull population consisted of purebred Holstein (H) individuals as well as H crossbred animals with various fractions of Brahman, Brown Swiss, Jersey, Red Dane, Red Sindhi, Sahiwal, and Thai Native [1]. Crossbred bulls accounted for 95% of the population, with most of them having a predominant H fraction and smaller fractions of other breeds. Further, all bulls in the population had H fractions ranging from 62.5% to 100%, with an average of 92%. The pedigree file encompassed a total of 304 animals, including bulls, sires, and dams.

The bulls were housed in open-barn stalls throughout the study period, except during semen collection, and were provided unrestricted access to mineral supplements, water, and fresh roughage. Concentrate feed (Charoen Pokphand Foods, Bangkok, Thailand) containing 16% crude protein, 2% fat, 14% fiber, and 13% moisture was administered to the bulls once daily. Fresh roughage comprised Guinea grass (*Panicum maximum*), Ruzi grass (*Brachiaria ruziziensis*), Napier grass (*Pennisetum purpureum*), and Para grass (*Brachiaria mutica*) harvested and transported to the bull stalls. Additionally, Guinea and Ruzi grass hay and silage were provided to the bulls during the dry season (November to June) when fresh grass was scarce.

### Traits

The traits were VOL (milliliters), NS (millions), and MOT (percentage). These traits were collected over a period spanning from October 2001 to July 2017 and were consistently evaluated by a single proficient technician throughout the duration of the research. Semen volume was quantified by measuring the amount of semen per ejaculate using a graduated tube. The NS per ejaculate was calculated by multiplying the semen volume (milliliters) by the sperm concentration (millions of sperm per milliliter). The determination of sperm concentration involved the utilization of a hematocytometer. The sperm concentration was derived by multiplying the average NS per counting area by a factor of 10,000 (NS per 0.1 milliliter) and subsequently by 100 (dilution ratio) to obtain the NS per milliliter. Sperm MOT was assessed by examining five microcells under an optical microscope with a magnification of 400×. Sperm MOT was defined as the mean value of two repeated measurements, representing the percentage of spermatozoa exhibiting forward movement. Bull identification, collection date and time, ejaculation number, ambient temperature (in degrees Celsius), and the name of the collector were recorded during each semen collection. For a comprehensive description of these traits, please refer to Sarakul et al [13].

### Genotypic data

**Figure 1:**
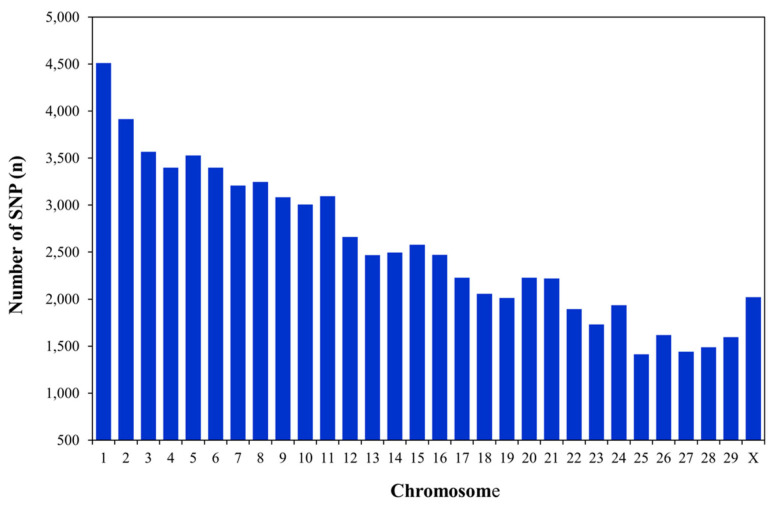
Total number of single nucleotide polymorphism per chromosome in the Thai multibreed dairy population.

The genotypic data came from semen samples collected from 61 out of the 131 bulls with available phenotypic records. Additionally, blood samples were obtained from 11 dams of sires [14]. Genomic DNA was extracted from frozen semen samples using the GenElute Mammalian Genomic DNA Miniprep kit (Sigma, Ronkonkoma, NY, USA) and from blood samples using the MasterPure DNA Purification kit (Epicentre Biotechnologies, Madison, WI, USA). The quality of the DNA samples was assessed using a NanoDrop 2000 spectrophotometer (Thermo Fisher Scientific Inc., Wilmington, DE, USA). DNA samples with a concentration of 15 ng/μL and an absorbance ratio of 1.8 at 260/280 nm were sent to GeneSeek for genotyping using GeneSeek Genomic Profiler (GGP) chips (GeneSeek Inc., Lincoln, NE, USA). Specifically, the 61 bulls were genotyped using GGP80K (76,694 SNP), whereas the 11 dams were genotyped using GGP9K (8,590 SNP). Dams genotyped with GGP9K were imputed to GGP80K [8] using version 2.2 of the FImpute program [15]. This imputation step utilized 2,661 animals genotyped with GGP9K (1,412 cows), GGP20K (570 cows), and GGP26K (540 cows), and GGP80K (89 sires and 50 cows) in a previous research project [14]. SNP genotypes from autosomes and the X chromosome with either a call rate lower than 0.90 or a minor allele frequency below 0.05 were excluded from the analysis. Thus, the edited genotype file had 76,519 actual and imputed SNP markers per animal. Due to limitations of the imputation program utilized for transitioning from low-density chips (GGP9K, GGP20K, and GGP26K) to a high-density chip (GGP80K), SNP markers from the Y chromosome were excluded during the construction of the input file. Figure 1 provides an overview of the total number of SNP markers per chromosome.

### Construction of the five single nucleotide polymorphism windows

**Figure 2:**
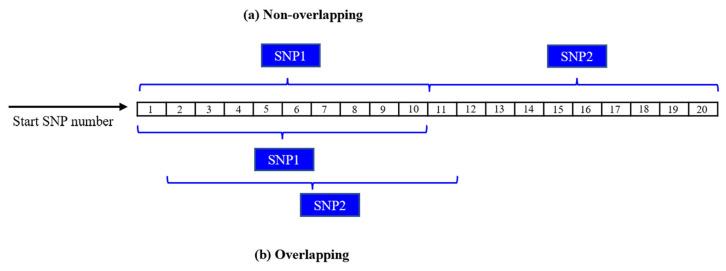
Sliding windows (a) non-overlapping and (b) overlapping.

Five SNP window sizes encompassing both overlapping and non-overlapping windows were used in this study. These five SNP windows contained one (SW1), ten (SW10), thirty (SW30), fifty (SW50), and one hundred (SW100) SNPs. The sizes of the overlapping windows were determined based on the contiguous number of SNPs within each window size. Thus, for SW10, the first window was constructed with SNP 1 to 10 (SNP_1_-SNP_2_-SNP_3_-SNP_4_-SNP_5_-SNP_6_-SNP_7_-SNP_8_-SNP_9_-SNP_10_), the second window encompassed SNP 2 to 11 (SNP_2_-SNP_3_-SNP_4_-SNP_5_-SNP_6_-SNP_7_-SNP_8_-SNP_9_-SNP_10_-SNP_11_), and so on. In contrast, the sizes of non-overlapping windows were determined by summing the number of SNPs within each window size. Thus, for SW10, the first window contained SNP 1 to 10 (SNP_1_-SNP_2_-SNP_3_-SNP_4_-SNP_5_-SNP_6_-SNP_7_-SNP_8_-SNP_9_-SNP_10_), the second window included SNP 11 to 20 (SNP_11_-SNP_12_-SNP_13_-SNP_14_-SNP_15_-SNP_16_-SNP_17_-SNP_18_-SNP_19_-SNP_20_), and so on. Figure 2 depicts the overlapping and non-overlapping windowing techniques utilized in this research.

### Genome-wide association analysis

The selection of five distinct window sizes (1, 10, 30, 50, and 100 SNPs) for both overlapping and non-overlapping windows, defined in terms of the values of SNP variances for all three semen traits, was based on the SNP variance values obtained through a GWAS (Wang et al [16]). The estimation of SNP variances was performed using a single-step genomic best linear unbiased prediction (GBLUP) procedure [17] implemented in program POSTGSF90 from the BLUPF90 family of programs [18].

A 3-trait genomic-polygenic repeatability model was employed to estimate the variance and covariance components among semen traits using restricted maximum likelihood. The estimation was performed with an average information algorithm implemented in the AIREMLF90 program [19]. Fixed effects in the model comprised contemporary group (year and month of semen collection), ejaculate order (first or second), age of the bull (months), ambient temperature (°C), and heterosis calculated as a function of heterozygosity. Heterozygosity was determined based on expected Holstein fraction in the sire × expected O fraction in the dam + expected O fraction in the sire × expected Holstein fraction in the dam, where O = other breeds (Brahman, Brown Swiss, Red Danish, Jersey, Red Sindhi, Sahiwal, and Thai Native [1]). Random effects were animal additive genetic, permanent environment, and residual. The mean of the animal additive genetic effects, permanent environment effects, and residual was assumed to be zero. The model in matrix notation can be represented as follows:


$$\text{y}=\text{Xb}+{\text{Z}}_{\text{a}}{\text{a}}_{\text{a}}+{\text{Z}}_{\text{p}}{\text{p}}_{\text{p}}+\text{e},$$

where **y** represents the vector of phenotypic records (VOL, NS, and MOT), ***b*** was a vector of fixed effects, ***a****_a_* was a vector of random animal additive genetic effects, ***p****_p_* was a vector of random permanent environmental effects, and ***e*** was a vector of random residuals. The incidence matrices ***X***, ***Z****_a_*, and ***Z****_p_* related records to fixed effects in vector ***b***, to random animal additive genetic effects in vector ***a****_a_*, and to random permanent environmental effects in vector ***p****_p_*, respectively. The mean of the animal additive genetic effects, permanent environment effects, and residual was assumed to be zero.

The variance-covariance matrix among animal additive genetic effects in the 3-trait genomic-polygenic repeatability model was defined as follows:


$$\text{V}ar\;\left[\begin{array}{c} a_{a}\\ pe\\ e \end{array}\right]=\left[\begin{array}{ccc} H⊗Va & 0 & 0\\ 0 & I⊗Vp_{p}, & 0\\ 0 & 0 & I⊗Ve \end{array}\right]$$

where ***H*** represented the genomic-polygenic relationship matrix, *V_a_* denoted a 3×3 matrix of additive genetic variances and covariances among VOL, NS, and MOT, and ⊗ was the Kronecker product. ***I*** was the identity matrix and *Vp_p_* represented a 3×3 matrix of permanent environment variances and covariances among three semen traits. Similarly, *Ve* was a 3×3 matrix of residual variances and covariances among semen traits.

The genomic-polygenic relationship matrix ***H*** [20] was calculated as follows:


$$H=\left[\begin{array}{cc} A_{11}+A_{12}A_{22}^{-1}(G_{22}-A_{22})A_{22}^{-1}A_{21} & A_{12}A_{22}^{-1}G_{22}\\ G_{22}\;A_{22}^{-1}A_{21} & G_{22} \end{array}\right],$$

where *A_11_* represents the matrix of additive genetic relationships among non-genotyped animals, *A_12_* denotes the matrix of additive relationships between non-genotyped and genotyped animals, *A_22_^−1^* is the inverse of the matrix of additive relationships among genotyped animals, and *G_22_* designates the matrix of genomic relationships among genotyped animals [20]. The matrix *G_22_* was constructed as follows:


$$G_{22}=ZZ^{\prime }/2\Sigma p_{j}(1-p_{j}),$$

where *p_j_* is the frequency of allele 2 in locus j, and *z_ij_* was defined as follows: (0–2*p_j_*) for the homozygous genotype 11 in locus j, (1–2*p_j_*) for the heterozygous genotypes 12 or 21 in locus j, and (2–2*p_j_*) for the homozygous genotype 22 in locus j [21,17]. The matrix *G_22_* was constructed using default weight factors (tau = 1, alpha = 0.95, beta = 0.05, gamma = 0, delta = 0, and omega = 1) and scaled using default restrictions (mean of diagonal elements of *G_22_* = mean of diagonal elements of *A_22_* and mean of off-diagonal elements of *G_22_* = mean of off-diagonal elements of *A_22_*) as defined by the PREGSF90 program of the BLUPF90 Family of Programs [18]. The proportion of the additive genetic variance explained by overlapping and non-overlapping SNP windows for SW1, SW10, SW30, SW50, and SW100 was determined using program POSTGSF90. The percentage of additive genetic variance explained by each SNP window was computed using the following formula:


$$\frac{Var(a_{i})}{\sigma_{a}^{2}}\times 100$$

where *Var*(*a_i_*) represents the additive genetic variance associated with the ith SNP window. For overlapping windows, *Var*(*a_i_*) was equal to the sum of the variances of all the contiguous SNP markers in the ith SNP window. For non-overlapping windows, *Var*(*a_i_*) was equal to the sum of the variances of all the SNP markers the ith SNP window. The term *σ_a_*^2^ represents the total additive genetic variance in the population. By applying this formula, we quantified the proportion of the additive genetic variance explained by each SNP window as a percentage of the total additive genetic variance. This measure provides information on the relative contribution of each SNP window to the total additive genetic variance of each semen trait in this study.

### Identification of SNP markers, genes, and pathway analysis

SNP markers that explained a minimum of 0.001% of the additive genetic variance for the three semen traits were selected to identify genes associated with VOL, NS, and MOT in both overlapping and non-overlapping windows. Thus, the minimum percentage of the additive genetic variance explained per overlapping and non-overlapping window was 0.001% for SW1, 0.01% for SW10, 0.03% for SW30, 0.05% for SW50, and 0.1% for SW100. The base pair (bp) positions of these SNP markers were used to locate genes or nearby genes in the UMD Bos taurus 3.1 assembly of the bovine genome database from the National Center for Biotechnology Information (NCBI) with R package Map2NCBI [22]. The pathway analysis included SNP markers that were located inside genes, within 2,500 bp, between 2,500 bp and 5,000 bp, between 5,000 bp and 25,000 bp, and more than 25,000 bp away from genes in the NCBI database.

Genes identified by the five overlapping and non-overlapping window sizes were used to identify biological pathways related to VOL, NS, and MOT. The Kyoto encyclopedia of genes and genomes database (KEGG) and the ClueGo plugin of Cytoscape [23] were employed for this analysis. The statistical test used for pathway analysis was a two-sided hypergeometric test, and multiple testing was corrected using the Bonferroni step-down procedure [24]. Biological pathways were considered significantly enriched or depleted for those traits if their p-values were lower than 0.05.

## RESULTS AND DISCUSSION

### Number of SNP and genes in overlapping and non-overlapping SNP windows of five sizes

Numbers of SNP markers accounting for at least 0.001% of the additive genetic variance for VOL, NS, and MOT in overlapping and non-overlapping windows for SW1, SW10, SW30, SW50, and SW100, identified by their distance from genes in the NCBI database, are presented in Table 1. The number of SNP explaining at least 0.001%, 0.01%, 0.03%, 0.05%, and 0.01% of the genetic variance in overlapping and non-overlapping windows represented 57% and 57% for SW1, 68% and 58% for SW10, 75 and 67% for SW30, 73% and 67% for SW50, and 71% and 65% for SW100, respectively. Large percentages of SNP markers associated with VOL, NS, and MOT were located inside genes (38.4%) and more than 25,000 bp away from genes (39.2%). Conversely, smaller percentages of SNP markers for the three semen traits were found within 2,500 bp (5.1%), between 2,500 and 5,000 bp (3.2%), and between 5,000 and 25,000 bp of genes (14.1%).

Overlapping and non-overlapping SNP windows contained a similar total number of SNP markers across all distances from genes in the NCBI database for SW1 (43,494 SNP vs 43,616 SNP). However, overlapping SNP windows included greater total numbers of SNP markers across all distances from genes than non-overlapping windows for SW10 (52,268 SNP vs 44,611 SNP), SW30 (56,620 SNP vs 50,996 SNP), SW50 (56,237 SNP vs 51,435 SNP), and SW100 (54,526 SNP vs 50,020 SNP). Total number of SNP markers across all distances from genes for overlapping windows were similar for SW30, SW50, and SW100. A similar situation existed for non-overlapping windows. These numbers of SNP markers close to genes indicate that SW1, SW10, and SW30 may be sufficient to determine pathway similarities among SNP sets.

Table 2 presents the numbers of genes associated with VOL, NS, and MOT identified by distance between SNP and gene in the NCBI database explaining at least 0.001% of the additive genetic variance in overlapping and non-overlapping SNP windows of five different sizes. On the average, 72% of genes associated with VOL, NS, and MOT in overlapping and non-overlapping SNP windows of five sizes were identified by SNP markers located inside genes or within 2,500 bp of genes. Seventeen percent of the genes were identified by SNP markers located between 2,500 and 5,000 bp from genes, 7% by SNP markers located between 5,000 and 25,000 bp from genes, and 4% by SNP markers located over 25,000 bp from genes. Thus, using only SNPs located inside genes or within 2,500 bp of genes in the NCBI database is enough to identify genes involved biological pathways affecting semen production traits in Thai cattle.

Numbers of genes associated with VOL, NS, and MOT identified by SNP from overlapping and non-overlapping SW1 were similar for all SNP-gene distances. Conversely, numbers of genes associated with these three semen traits identified by SNP from overlapping were higher than those from non-overlapping for SW10, SW30, SW50, and SW100 for all SNP-gene distances. The similarities observed among numbers of genes identified through overlapping and non-overlapping SW30, SW50, and SW100 indicate that SW30 may be sufficient to determine pathway associations affecting. It is important to note that no previous studies comparing overlapping and non-overlapping SNP windows, specifically considering SW1, SW10, SW30, SW50, and SW100 were found in the literature. These findings indicate that overlapping windows yielded a greater amount of genetic information than non-overlapping windows across all five window sizes. These differences may be due to the methods used to tally numbers of SNP in overlapping and non-overlapping windows. In the case of overlapping windows, the analysis involved multiple consecutive SNPs, whereas non-overlapping windows were created by summing the number of SNPs within each window size. These methodological differences influenced the identification of both the number of SNPs and the genes whose alleles are likely inherited together. However, research in livestock and humans found that haplotype analyses were superior to individual SNP analyses [25–28]. Further, a study involving Nelore cattle [5] found that haplotypes of five different sizes (SW3, SW5, SW7, SW9, SW11) detected more QTLs than single SNPs. Lastly, research on human diseases reported that overlapping windows performed better than non-overlapping windows [29].

### Biological pathways in overlapping and non-overlapping SNP windows of five sizes

All genes determined to be associated with VOL, NS, and MOT using SNP markers from overlapping and non-overlapping SW, SW10, SW30, SW50, and SW100 (Table 2) were utilized to identify biological pathways in the Thai multibreed dairy population. This analysis utilized *Bos taurus* information from the KEGG database and the ClueGo plugin of Cytoscape [23].

Table 3 presents the number of biological pathways and the number of genes within those pathways in common across overlapping and non-overlapping SW1, SW10, SW30, SW50, and SW100 for VOL, NS, and MOT using *Bos taurus* information from the KEGG database. Table 3 shows that the number of shared biological pathways and genes within pathways were lower for overlapping SW1 (8 pathways and 904 genes) than for non-overlapping SW1 (9 pathways and 1,049 genes). Conversely, the number of shared biological pathways and genes within pathways were higher for overlapping than non-overlapping SW10 (9 pathways and 1,052 genes vs 5 pathways and 533 genes), SW30 (11 pathways and 1,343 genes vs 7 pathways and 1,048 genes), SW50 (10 pathways and 1,351 genes vs 8 pathways and 1,096 genes), and SW100 (10 pathways and 1,327 genes vs 6 pathways and 817 genes). These results showed that overlapping SNP windows consistently yielded a higher number of shared biological pathways and genes than non-overlapping SNP windows, indicating that overlapping windows should be preferred to capture genetic interactions and regulatory mechanisms for the three semen traits in the Thai multibreed dairy population. These sets of SNPs are associated with genes that influence the modulation of biological pathways affecting semen traits, thus they could be incorporated into customized genotyping chips. This would enhance the accuracy of genomic selection for semen production traits in Thailand.

Among overlapping windows, the highest number of biological pathways associated with VOL, NS, and MOT was observed in SW30 (11 pathways), followed by SW50 (10 pathways), 100 (10 pathways), SW10 (9 pathways), and SW1 (8 pathways). Among non-overlapping windows, SW1 captured the most comprehensive set of pathways contributing to VOL, NS, and MOT (9 pathways), followed by SW50 (8 pathways), SW30 (7 pathways), SW100 (6 pathways), and SW10 (5 pathways). The biological pathways involving genes associated with semen traits are shown in Supplementary Table 1 for overlapping windows and Supplementary Table 2 for non-overlapping windows.

Similarly, the highest number of genes within shared biological pathways associated with VOL, NS, and MOT were observed in overlapping SW50 (1,351 genes), followed by SW30 (1,343 genes), SW100 (1,327 genes), SW10 (1,052 genes), and SW1 (904 genes). Conversely, for non-overlapping windows, the largest number of genes within shared pathways influencing VOL, NS, and MOT occurred in SW50 (1,096 genes), followed by SW1 (1,049 genes), SW30 (1,048 genes), SW100 (817 genes), and SW10 (533 genes). Thus, overlapping SNP windows in general, and SW50 in particular, were the most effective to capture genes within shared pathways contributing to the three semen traits, highlighting the potential biological relevance of these genes to VOL, NS, and MOT. Conversely, non-overlapping SNP windows identified smaller number genes within shared pathways affecting VOL, NS, and MOT, particularly SW10. These numbers indicate that overlapping SNP windows in general, and SW30 and SW50 in particular, should be preferred to maximize the identification of pathways and genes.

Findings here emphasize the importance of evaluating overlapping and non-overlapping windows of various sizes when determining biological pathways and genes associated with complex traits. Although medium sized overlapping windows (SW30 and SW50) were the most effective to capture a broader range of shared pathways and genes, this may not be the case in other populations; hence the need for assessing the effectiveness of various overlapping and non-overlapping window sizes in each population before choosing one for widespread use.

In conclusion, this study yielded valuable insights into the genetic basis of VOL, NS, and MOT by assessing five sets of biological pathways and genes. We identified a substantial number of SNP markers that are either within or near genes associated with semen traits. Overlapping windows consistently identified a greater number of shared biological pathways and genes than non-overlapping windows. Knowledge of these shared pathways and genes enhance our understanding of the biological processes involved in these traits and contribute to the development of increasingly more effective strategies for genetic improvement of semen traits in the Thai multibreed dairy population.

## Figures and Tables

**Table 1:** Number of SNP for semen traits explaining at least 0.001% of the additive genetic variance in overlapping and non-overlapping windows of five sizes

Window size^1)^	Type	Distance between SNP and gene (bp)					Total

		Inside gene	(1 to 2,500)	(2,500 to 5,000)	(5,000 to 25,000)	>25,000	
SW 1	Overlap	16,677	2,229	1,434	6,098	17,056	43,494
	Non-overlap	16,724	2,240	1,437	6,113	17,102	43,616
SW 10	Overlap	20,134	2,703	1,675	7,439	20,317	52,268
	Non-overlap	17,282	2,329	1,435	6,298	17,267	44,611
SW 30	Overlap	21,738	2,932	1,834	8,038	22,078	56,620
	Non-overlap	19,604	2,677	1,683	7,190	19,842	50,996
SW 50	Overlap	21,650	2,916	1,833	7,944	21,894	56,237
	Non-overlap	19,793	2,634	1,679	7,236	20,093	51,435
SW 100	Overlap	20,855	2,747	1,765	7,561	21,598	54,526
	Non-overlap	19,168	2,528	1,635	7,026	19,663	50,020

SNP, single nucleotide polymorphism.

1) SW1, SW10, SW30, SW50, SW100 means either overlapping or non-overlapping window size of 1, 10, 30, 50, 100 SNP.

**Table 2:** Number of genes for semen traits explaining at least 0.001% of the additive genetic variance in overlapping and non-overlapping windows of five sizes

Window size^1)^	Type	Distance between SNP and gene (bp)					Total

		Inside gene	(1 to 2,500)	(2,500 to 5,000)	(5,000 to 25,000)	>25,000	
SW 1	Overlap	6,692	1,563	840	1,882	422	11,399
	Non-overlap	6,706	1,569	841	1,884	425	11,425
SW 10	Overlap	8,040	1,838	969	2,346	498	13,691
	Non-overlap	6,911	1,611	819	1,997	444	11,782
SW 30	Overlap	8,596	2,009	1,067	2,522	534	14,728
	Non-overlap	7,784	1,845	970	2,292	505	13,396
SW 50	Overlap	8,610	2,007	1,065	2,490	529	14,701
	Non-overlap	7,889	1,823	981	2,288	486	13,467
SW 100	Overlap	8,209	1,878	1,024	2,401	528	14,040
	Non-overlap	7,543	1,730	953	2,244	488	12,958

SNP, single nucleotide polymorphism.

1) SW1, SW10, SW30, SW50, SW100 means either overlapping or non-overlapping window size of 1, 10, 30, 50, 100 SNP.

**Table 3:** Number of biological pathways (NP) and number of genes in biological pathways (NG) in common across overlapping and non-overlapping SNP windows of five sizes for semen traits

Window size^1)^	Type	SW1		SW10		SW30		SW50		SW100	
				
		NP	NG	NP	NG	NP	NG	NP	NG	NP	NG
SW 1	Overlap	**8**	**904**								
	Non-overlap	**9**	**1,049**								
SW 10	Overlap	4	421	**9**	**1,052**						
	Non-overlap	2	280	**5**	**533**						
SW 30	Overlap	5	511	6	364	**11**	**1,343**				
	Non-overlap	6	524	2	336	**7**	**1,048**				
SW 50	Overlap	4	505	5	635	10	1021	**10**	**1,351**		
	Non-overlap	6	503	2	337	6	726	**8**	**1,096**		
SW 100	Overlap	5	494	4	591	7	860	7	889	**10**	**1,327**
	Non-overlap	4	409	2	334	5	625	6	664	**6**	**817**

1) SW1, SW10, SW30, SW50, SW100 means either overlapping or non-overlapping window size of 1, 10, 30, 50, 100 SNP.

## Data Availability

The data used in this study will be shared upon a reasonable request to the corresponding author.

## SUPPLEMENTARY MATERIAL

**Supplementary Table S1**. Biological pathways involving genes associated with semen traits when utilizing overlapping SNP windows of five sizes

**Supplementary Table S2**. Biological pathways involving genes associated with semen traits when utilizing non-overlapping SNP windows of five sizes
