# Supplementary material for: A comparison of five sets of overlapping and non-overlapping sliding windows for semen production traits in the Thai multibreed dairy population
Source: Anim Biosci. 2023 Nov 1;37(3):428–36. doi: 10.5713/ab.23.0230 (PMC10915195; doi:10.5713/ab.23.0230)
Supplement: Supplementary file 1 [file ab-23-0230-Supplementary-Table-S1.pdf]

Supplementary Table S1. Biological pathways involving genes associated with semen traits when utilizing overlapping SNP windows of five sizes

Window size[1]	Name of pathway[2]	P-value[3]	Percent genetic variance	Number of genes (n)
SW1	Olfactory transduction	2.6×10^{-14}	0.90	302
	Ribosome	0.0001	0.14	32
	Protein digestion and absorption	0.0005	0.17	65
	Oxytocin signaling pathway	0.0100	0.33	82
	Calcium signaling pathway	0.0181	0.36	99
	Regulation of actin cytoskeleton	0.0182	0.45	113
	Focal adhesion	0.0311	0.42	104
	Rap1 signaling pathway	0.0352	0.40	107
SW10	Olfactory transduction	4.4×10^{-13}	12.78	356
	Ribosome	2.1×10^{-6}	0.97	38
	cGMP-PKG signaling pathway	0.0007	2.99	104
	Regulation of actin cytoskeleton	0.0078	3.54	123
	MAPK signaling pathway	0.0079	5.40	168
	Antigen processing and presentation	0.0116	1.00	23

	Wnt signaling pathway	0.0125	3.43	92
	Phagosome	0.0399	1.38	56
	Oxytocin signaling pathway	0.0402	3.87	92
SW30	Olfactory transduction	1.6×10^{-12}	25.36	409
	Calcium signaling pathway	3.4×10^{-5}	11.93	131
	Ribosome	4.3×10^{-5}	3.10	48
	MAPK signaling pathway	0.0007	17.33	185
	Antigen processing and presentation	0.0027	1.00	25
	Hippo signaling pathway	0.0036	10.55	102
	Wnt signaling pathway	0.0043	8.45	100
	Axon guidance	0.0062	16.74	114
	Focal adhesion	0.0064	9.62	129
	Phospholipase D signaling pathway	0.0153	9.73	100

Supplementary Table S1 (Continued)

Window size[1]	Name of pathway[2]	P-value[3]	Percent genetic variance	Number of genes (n)
SW50	Olfactory transduction	7.7×10^{-15}	27.79	426
	Ribosome	1.8×10^{-5}	5.06	47
	Hippo signaling pathway	0.0003	16.69	105
	Calcium signaling pathway	0.0007	19.43	127
	Focal adhesion	0.0008	21.63	132
	Antigen processing and presentation	0.0074	5.54	26
	MAPK signaling pathway	0.0111	27.78	180
	Axon guidance	0.0181	17.02	112
	Phospholipase D signaling pathway	0.0309	16.03	99
	Wnt signaling pathway	0.0372	13.91	97
SW100	Olfactory transduction	1.2×10^{-15}	32.08	400
	Ribosome	1.6×10^{-5}	8.63	43
	Calcium signaling pathway	0.0002	38.60	124

	Rap1 signaling pathway	0.0071	38.59	129
	Focal adhesion	0.0115	40.97	123
	Platelet activation	0.024	25.06	77
	Wnt signaling pathway	0.0246	29.79	94
	Hippo signaling pathway	0.0301	31.93	95
	MAPK signaling pathway	0.0363	52.99	170
	Glutamatergic synapse	0.0496	20.93	72

[1] SW1, SW10, SW30, SW50, SW100 means either overlapping or non-overlapping window size of 1, 10, 30, 50, 100 SNP.

[2] Kyoto encyclopedia of genes and genomes (KEGG) database.

[3] Bonferroni step down.
