# Supplementary material for: A comparison of five sets of overlapping and non-overlapping sliding windows for semen production traits in the Thai multibreed dairy population
Source: Anim Biosci. 2023 Nov 1;37(3):428–36. doi: 10.5713/ab.23.0230 (PMC10915195; doi:10.5713/ab.23.0230)
Supplement: Supplementary file 2 [file ab-23-0230-Supplementary-Table-S2.pdf]

511 **Supplementary Table S2.** Biological pathways involving genes associated with semen traits when utilizing non-overlapping SNP windows of five sizes

Window size[1]	Name of pathway[2]	P-value[3]	Percent genetic variance	Number of genes (n)
SW1	Olfactory transduction	1.3×10^{-14}	1.01	302
	Ribosome	0.0001	0.14	32
	Regulation of actin cytoskeleton	0.0003	0.44	113
	Focal adhesion	0.0098	0.37	105
	Protein digestion and absorption	0.0104	1.69	65
	Oxytocin signaling pathway	0.0192	0.30	82
	Rap1 signaling pathway	0.0265	0.37	108
	Calcium signaling pathway	0.0324	0.35	99
	MAPK signaling pathway	0.0498	0.58	143
SW10	Olfactory transduction	2.3×10^{-14}	9.70	325
	Ribosome	0.0003	0.98	35
	Antigen processing and presentation	0.0028	0.74	17
	cGMP-PKG signaling pathway	0.0044	4.12	92

	Glutamatergic synapse	0.0343	2.52	64
SW30	Olfactory transduction	6.0×10^{-16}	17.62	379
	Ribosome	0.0001	2.77	42
	Focal adhesion	0.0003	11.43	125
	MAPK signaling pathway	0.0026	14.35	170
	Calcium signaling pathway	0.0027	10.70	117
	Wnt signaling pathway	0.0164	7.70	92
	Rap1 signaling pathway	0.0291	8.49	123

513 **Supplementary Table S2.** (Continued)

Window size[1]	Name of pathway[2]	P-value[3]	Percent genetic variance	Number of genes (n)
SW50	Olfactory transduction	1.0×10^{-19}	5.86	362
	Ribosome	0.0023	26.23	46
	MAPK signaling pathway	0.0036	24.50	170
	Focal adhesion	0.0059	19.17	121
	Hippo signaling pathway	0.0069	15.29	95
	Wnt signaling pathway	0.0084	13.59	93
	Calcium signaling pathway	0.0106	16.71	115
	Oxytocin signaling pathway	0.0142	15.65	94
SW100	Olfactory transduction	5.0×10^{-22}	56.82	369
	Ribosome	0.0004	9.66	41
	Calcium signaling pathway	0.0065	32.94	112
	Focal adhesion	0.0074	37.24	116
	Wnt signaling pathway	0.0074	25.09	90

Hippo signaling pathway	0.0369	27.36	89

[1]SW1, SW10, SW30, SW50, SW100 means either overlapping or non-overlapping window size of 1, 10, 30, 50, 100 SNP.

[2]Kyoto Encyclopedia of Genes and Genomes (KEGG) database.

[3]Bonferroni step down.
